# Loss of survival advantage for deficient mismatch repair in patients with advanced colorectal cancer may be caused by changes in prognostic value of CD8+T cell

**DOI:** 10.1186/s12957-020-01970-0

**Published:** 2020-08-07

**Authors:** Bingyan Wang, Fei Li, Limei Guo, Siyi Lu, Junren Ma, Yanpeng Ma, Yan Meng, Junwei Wang, Xin Zhou, Wei Fu

**Affiliations:** 1 Department of General Surgery, Peking University Third Hospital, Beijing, China; 2 Department of Pathology, School of Basic Medical Sciences, Third Hospital, Peking University Health Science Center, Beijing, China

**Keywords:** Colorectal cancer, Deficient mismatch repair, Tumor-infiltrating lymphocyte, PD-L1, Prognosis

## Abstract

**Background:**

Patients with stage II deficient mismatch repair (dMMR) show a better prognosis than patients with colorectal cancer (CRC) with proficient mismatch repair (pMMR). However, this beneficial effect is decreased in advanced stages of the disease. This study was conducted to investigate the prognostic value of dMMR in different stage and alterations in the tumor microenvironment.

**Methods:**

This was a matched retrospective cohort study. Thirty-two patients with stage III&IV dMMR matched with 32 patients with stage I&II dMMR and 64 patients with pMMR were evaluated. Immunohistochemistry analysis was performed for the 64 patients with dMMR to explore the expression and prognostic effect of CD3, CD4, CD8, and PD-L1.

**Results:**

Patients with stage III–IV dMMR showed no advantage in overall survival (OS) and disease-free survival (DFS) compared to patients with pMMR *(P* = 0.244, *P* = 0.667). No expression differences in CD3, CD4, CD8, and PD-L1 at the center of the tumor (CT) or invasive margin (IM) were found between patients with stage I&II and stage III&IV dMMR. High CD3 expression at the CT and high CD3 an CD4 expression at the IM improved both OS and DFS. High CD8 expression showed opposite prognostic value in patients with stage I&II and III&IV dMMR. A similar tendency was observed for PD-L1 expression.

**Conclusion:**

Patients with stage III–IV dMMR showed no prognostic advantage over patients with pMMR. Expression of CD3, CD4, CD8, and PD-L1 was similar between stage I&II and III&IV dMMR CRC. High CD3 expression at the CT and high CD3 and CD4 expression at the IM can significantly improve patient prognosis. The opposite prognostic tendency of CD8 and PD-L1 for patients with stage I&II and III&IV dMMR may be relevant to CD8+T cell exhaustion and functional changes at inhibitory immune checkpoints.

## Background

Colorectal cancer (CRC) is the third most common malignancy worldwide [1]. The Cancer Genome Atlas classification [2] and Consensus Molecular subtype classification [3] both define a subgroup of patients with deficient mismatch repair (dMMR) and show microsatellite instability high (MSI-H). MSI is the molecular fingerprint of dMMR [4]. Pathological features for dMMR CRC are typically associated with poor differentiation and increased tumor-infiltrating lymphocytes (TILs) [4]. The National Comprehensive Cancer Network guidelines [5] state that patients with stage II MSI-H have a better prognosis and do not benefit from fluorouracil adjuvant therapy. However, whether patients with dMMR show a survival advantage in advanced CRC remains controversial. Several randomized controlled trials revealed no advantage [6–8] or worse [9] survival of patients with stage III or IV dMMR. Our previous meta-analysis showed no obvious survival benefit for patients with dMMR in an advanced stage [10].

Tumors can express antigens, known as tumor-associated antigens (TAAs), which trigger immune responses [11, 12]. Patients with dMMR present higher levels of TAAs and increased TILs than patients with proficient MMR (pMMR) [4, 13]. Many studies have shown that TIL density is closely related to tumor prognosis [14, 15]. This may explain why patients with MSI-H show better prognosis. However, lymph node or distal metastasis indicate immune escape of cancer [16]. Immune escape is associated with T cell exhaustion and upregulation of inhibitory checkpoint molecules such as PD-1/PD-L1 [17]. This may be related to the loss of beneficial effect for patients with dMMR at an advanced stage.

This study was conducted to explore the prognostic value of dMMR in patients with advanced CRC and whether expression or prognostic differences in CD3, CD4, CD8, and PD-L1 exist between patients with early and advanced dMMR CRC.

## Methods

### Patient selection

From 2010 to 2018, 1460 patients diagnosed with CRC underwent radical surgical treatment at our hospital. Basic information was retrieved using the electrical medical record system.

MMR status was judged according to the pathological reports from the pathology department of our hospital which tested MLH1, MSH2, MSH6, and PMS2 through immunohistochemistry(IHC) method. Negativity for any of the four markers was considered to indicate a dMMR status. All dMMR results were confirmed by a pathologist in our hospital.

**Fig. 1:**
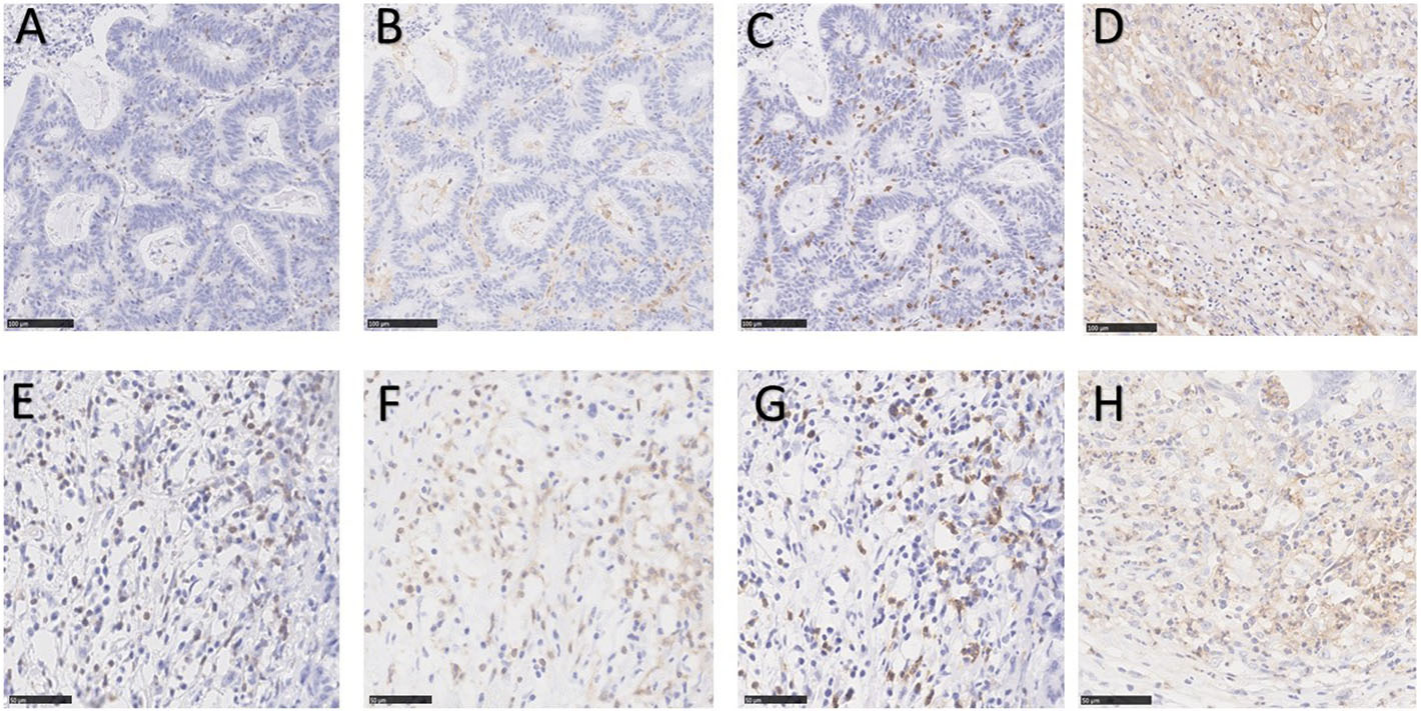
Example of immunohistochemical staining of CD3, CD4, CD8, and PD-L1. **a**–**d** CD3, CD4, CD8, and positive PD-L1 expression at center of tumor; **e**–**h** CD3, CD4, CD8, and positive PD-L1 expression at invasive margin

After screening, a total of 32 patients with stage III&IV dMMR were available for further analysis. Thirty-two patients with stage I&II dMMR and 64 patients with pMMR were matched using propensity score for further analysis (Fig. 1).

### Immunohistochemistry

CD3 (ab699, Abcam), CD4 (ab133616, Abcam), CD8 (ab93278, Abcam), and PD-L1 ([SP142]-C-terminal, prediluted, Abcam) were used to test expression of the corresponding proteins. Tissue sections 5-μm-thick were deparaffinized and dehydrated. Endogenous peroxidase was blocked with 3% hydrogen peroxide for 10 min at room temperature. Antigen retrieval was performed in Ethylene Diamine Tetraacetic Acid (EDTA), for 2 min at 100 °C. The slides were incubated with the primary antibody at 37 °C for 2 h. After three washes with phosphate-buffered saline, the slides were co-incubated with horseradish peroxidase-labelled goat anti rabbit/mouse secondary antibodies. The slides were counter-stained with hematoxylin. Each slide was examined by an experienced pathologist to obtain the coincident immunohistochemical results.

### Image analysis and data synthesis

All slides were digitalized using NanoZoomer (Hamamatsu Photonics, Hamamatsu, Japan). The center of the tumor (CT) and invasive margin (IM) were drawn by an experienced pathologist, and three non-adjacent areas were randomly chosen to evaluate both the CT and IM. The pathologist who selected the areas of interest was blinded to the patients’ information. The density (n/mm^2^) of CD3/CD4/CD8+T cells at the CT or IM was counted using Image-Pro Plus 6.0 (Media Cybernetics, Rockville, MD, USA) software. The results of the three regions were averaged and statistically analyzed (Fig. 1).

The cut-off values for CD3, CD4, and CD8 were obtained from receiver operating characteristic (ROC) curves were drawn for each group in relation to disease-specific mortality. The immunoscore (IS) point was counted according to the immunoscore classification proposed by Galon et al. [18]. The IS was generated from four points: CT and invasive margin (IM) for CD3 and CD8. High expression of each region was scored as 1 point. IS0-2 and IS 3-4 were considered as IS-low and IS-high. Tissues were considered as PD-L1-positive when more than 5% of tumor cells or TILs showed medium or strong staining (Fig. 1) [19, 20].

### Statistical analysis

Statistical analyses were conducted using SPSS 24.0 software (SPSS, Inc., Chicago, IL, USA). Data normality was determined using the Kolmogorov-Sminov method. Normally and non-normally distributed data are expressed as the mean ± SD deviation and median (quartile spacing). Differences between groups were verified by independent sample *t* test or Mann-Whitney *U* test according to the normality result. Dichotomous variables were analyzed by Fisher’s exact test.

The survival curve was drawn by the Kaplan-Meier method. Multivariate analysis was performed using Cox regression, and predictive values were measured using the hazard ratio (HR) and 95% confidence interval (CI). The samples were matched at 1:1 using the SPSS propensity score module. All tests were two-sided, and *P* < 0.05 was considered as statistically significant.

## Results

### Pathological and survival information

Thirty-two patients with III&IV dMMR, 32 patients with stage I&II dMMR, and 64 propensity score-matched patients with pMMR were included in pathological and survival analyses.

For patients with dMMR, there were no significant differences between groups in age (*P* = 0.987), body mass index (BMI) (*P* = 0.614), tumor location (*P* = 0.805), positive tumor deposit (*P* = 0.148), perineural evasion (PNI) (*P* = 0.277), tumor differentiation (*P* = 0.486), length of stay (*P* = 0.770), and follow-up time (*P* = 0.151) (Table 1).

**Table 1:** Clinical and pathological information for patients with dMMR and matched patients with pMMR

Group	Stage I&II dMMR (*n* = 32)	Stage III&IV dMMR (*n* = 32)	*P* value (I&II vs. III&IV)	Stage I-IV pMMR (*n* = 64)	*P* value (dMMR vs. pMMR)
Age(year)	62.66 ± 13.85	62.59 ± 17.35	0.987	62.84 ± 12.34	0.592
Sex, *n* (%)			0.614		1.000
Male	17 (53.1%)	19 (59.4%)		36 (56.3%)	
Female	15 (46.9%)	13 (40.6%)		28 (43.8%)	
BMI (kg/m^2^)	23.84 ± 3.92	22.87 ± 3.15	0.280	24.44 (21.78–25.68)	0.475
Tumor location			0.805		1.000
Right-sided	19 (59.4%)	21 (65.6%)		40 (62.5%)	
Left-sided	6 (18.8%)	6 (18.8%)		12 (18.8%)	
Rectum	7 (21.9%)	5 (15.6%)		12 (18.8%)	
UICC-TNM stage			/		1.000
I	8 (25%)			8 (12.5%)	
II	24 (75%)			24 (37.5%)	
III		28 (87.5%)		28 (43.8%)	
IV		4 (12.5%)		4 (6.2%)	
Tumor deposit			0.148		0.826
Negative	30 (93.8%)	25 (78.1%)		54 (84.4%)	
Positive	2 (6.2%)	7 (21.1%)		10 (15.6%	
PNI			0.277		0.219
Negative	29 (90.6%)	26 (81.3%)		48 (75.0%)	
Positive	3 (9.4%)	6 (18.8%)		16 (25.0%)	
Follow-up time	51.14 (28.80–79.43)	41.03 (19.24–61.76)	0.151	49.26 (28.73–69.20)	0.343
Length of hospital stay	18.38 (11.25–25.5)	19.09 (13–25.5)	0.770	16.22 (13–18)	0.550
Differentiation			0.486		0.046
High	1 (3.1%)	0		2 (3.1%)	
Medium	19 (59.4%)	17 (53.1%)		48 (75.0%)	
Low	12 (37.5%)	15 (46.9%)		14 (21.9%)	

*BMI* body mass index, *UICC* Union for International Cancer Control, *PNI* perineural invasion

We compared all 64 patients with dMMR and 64 propensity score-matched patients with pMMR. No difference was found in BMI (*P* = 0.475), tumor deposit (*P* = 0.826), PNI (*P* = 0.219), follow-up time (*P =* 0.343), or length of hospital stay (*P* = 0.550). Additionally, patients with pMMR showed a higher proportion of poorly differentiated tumors (*P* = 0.046) (Table 1).

**Fig. 2:**
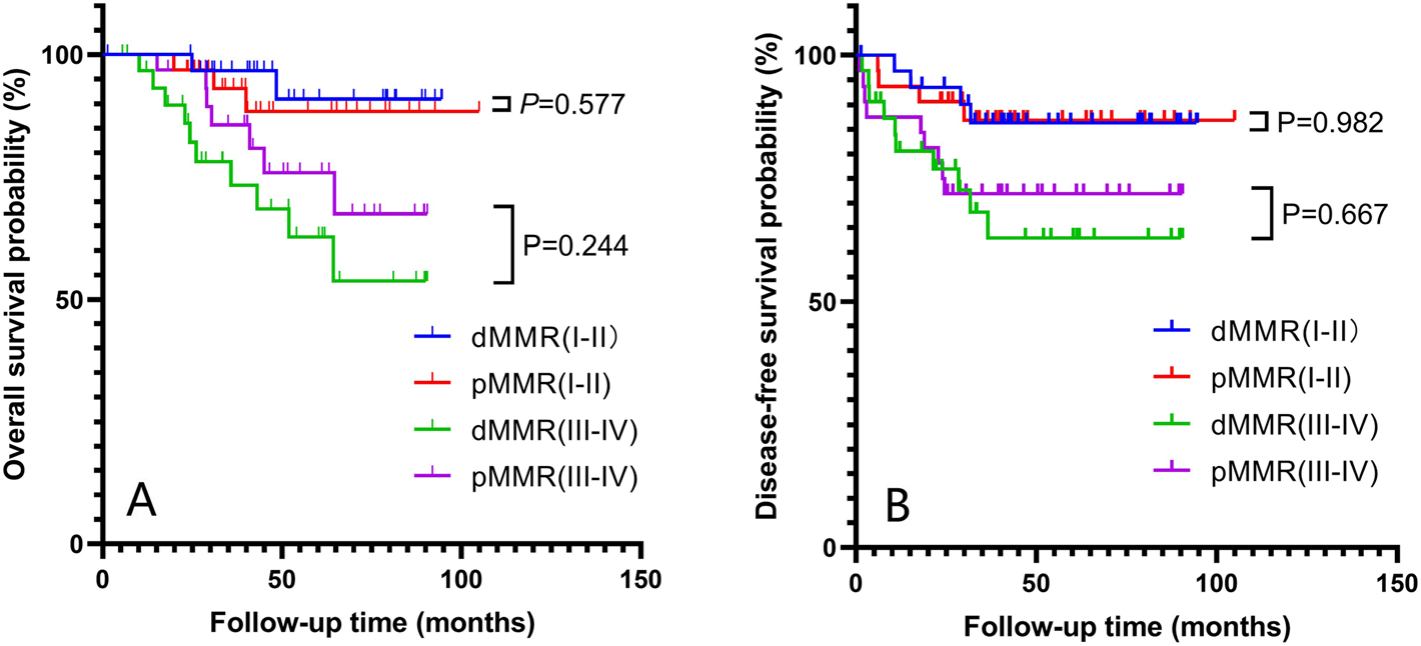
Kaplan-Meier survival curves of overall survival (**a**) and disease-free survival (**b**) for all included patients

The Kaplan-Meier revealed no significant difference between the MMR status for overall survival (OS) and disease-free survival (DFS) in patients with stage I&II (*P* = 0.577, *P* = 0.982) and III&IV (*P* = 0.244, *P* = 0.667) (Fig. 2).

### CD3, CD4, CD8, and PD-L1 expression and survival analysis

For patients with dMMR, no expression differences at the CT were detected for the density of CD3 (44.69, IQR 13.38–61.35 vs. 29.88, IQR 9.31–44.27; *P* = 0.210), CD4 (39.99, IQR 20.50–52.10 vs. 30.64, IQR 17.06–39.33; *P* = 0.098), and CD8 (35.63, IQR 8–33–44.19 vs. 22.65, IQR 5.96–33.44; *P* = 0.587) and positive PD-L1 rate (31.3% vs. 40.6%, *P* = 0.434) between patients with stage I&II and III&IV.

**Fig. 3:**
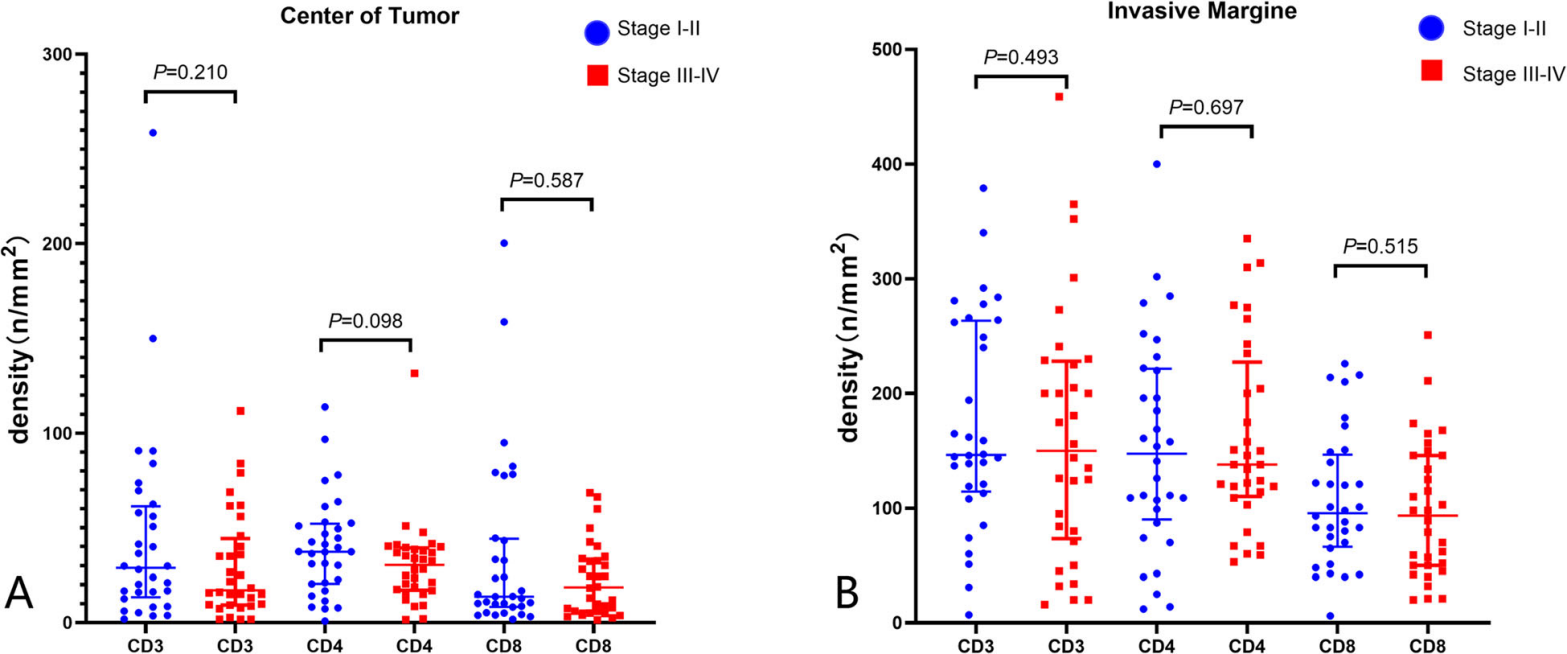
Expression of CD3, CD4, and CD8 density between stages I–II and stage III–IV dMMR patients at center of tumor (**a**) and invasive margin (**b**)

Similar negative results were found in the IM. The density of CD3 (177.33, IQR 114.50–263.50 vs. 162.28, IQR 73.25–228.00; *P* = 0.493), CD4 (154.25, IQR 90.00–221.50 vs. 161.38, IQR 110.25–227.25; *P* = 0.697), CD8 (101.38, IQR 58.25–148.25 vs. 103.97, IQR 48.50–138.50; *P* = 0.515), and positive PD-L1 expression rate (62.5% vs. 56.3%, *P* = 0.611) between patients with stage I&II and III&IV showed no significant differences (Fig. 3).

Kaplan-Meier survival analysis was performed for OS and DFS based on the high/low expression of CD3, CD4, and CD8 and positive/negative expression of PD-L1 at the CT or IM. The prognostic value of IS was also explored.

**Fig. 4:**
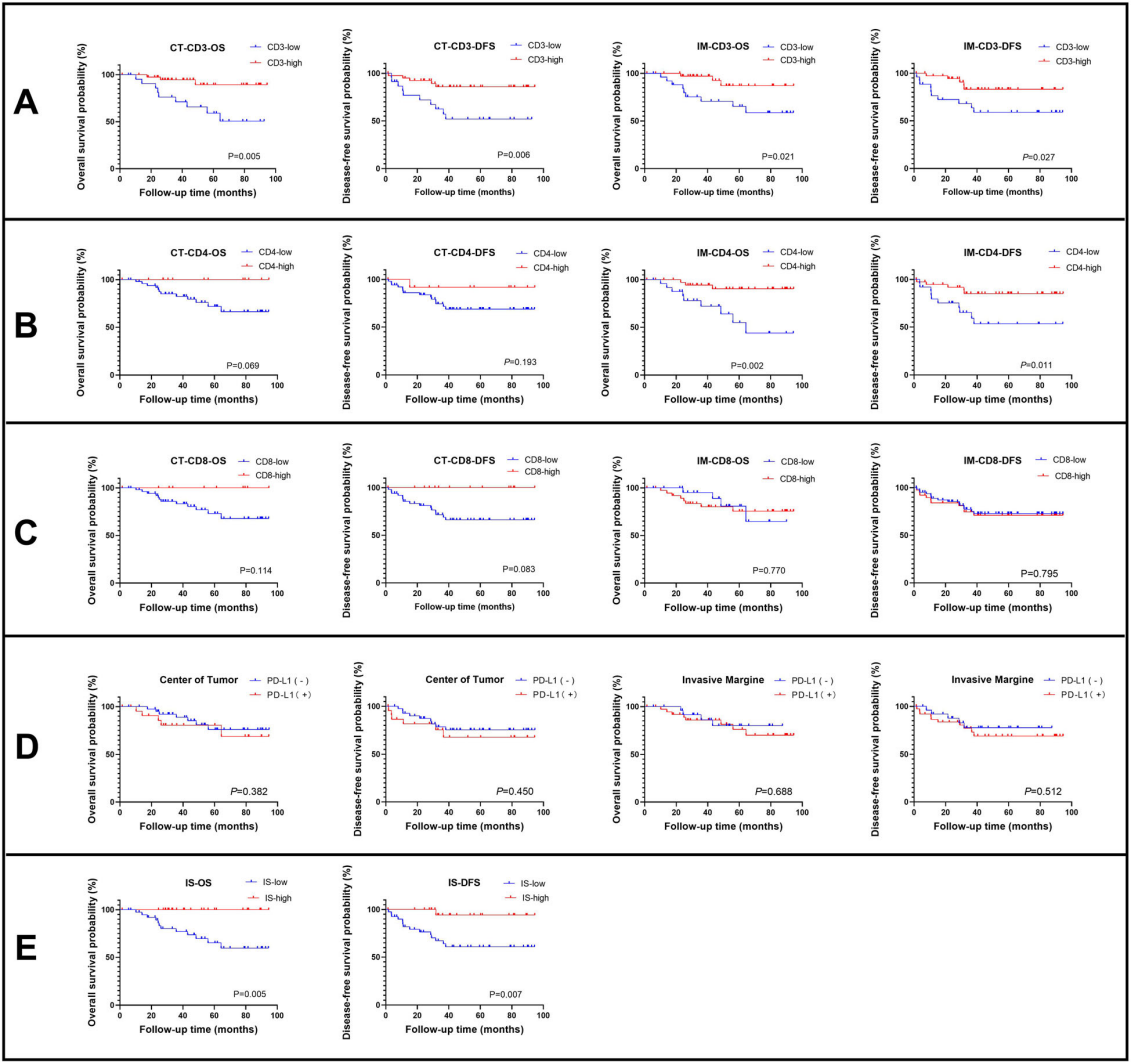
Kaplan-Meier curve of overall survival and disease-free survival for high/low expression of CD3 (**a**), CD4 (**b**), CD8 (**c**), and positive/negative expression of PD-L1 (**d**) at center of tumor and invasive margin as well as high/low immunoscore (**e**)

The results showed that for all patients with dMMR, high CD3 expression at both the CT and IM improved OS (*P* = 0.005, *P* = 0.021) and DFS (*P* = 0.006, *P* = 0.027). High CD4 expression at the IM improved OS (*P* = 0.002) and DFS (*P* = 0.011). A high IS improved both OS (*P* = 0.005) and DFS (*P* = 0.007). The expression level of CD8 at the CT and IM showed no significant influence on OS (*P* = 0.014, *P* = 0.770) or DFS (*P* = 0.083, *P* = 0.795). PD-L1 expression at the CT or IM also showed no obvious influence on OS (*P* = 0.382, *P* = 0.688) or DFS (*P* = 0.450, *P* = 0.512) (Fig. 4).

Multivariate analysis was performed by Cox regression to further explore the independent risk factors for survival. The results showed that high expression of CD3 at the CT (*P* = 0.012) and high expression of CD3 and CD4 at the IM (*P* = 0.034, *P* = 0.001) were independent beneficial factors for OS. High expression of CD3 at the CT (*P* = 0.011) and high expression of CD3 and CD4 at the IM (*P* = 0.006, *P* = 0.001) as well as high IS (*P* = 0.026) were independent beneficial factors for DFS (Table 2).

**Table 2:** Univariate and multivariate survival analysis for risk factors for overall survival and disease-free survival

	Univariate for OS	Multivariate for OS		Univariate for DFS	Multivariate for DFS	
Factors	*P*	HR (95% CI)	*P*	*P*	HR (95% CI)	*P*
CT-CD3(H/L)	0.005	0.026 (0.050–0.685)	0.012	0.006	0.217 (0.067–0.700)	0.011
IM-CD3(H/L)	0.091	0.242 (0.065–0.899)	0.034	0.027	0.188 (0.058–0.617)	0.006
CT-CD4(H/L)	0.069	—	0.261	0.192	—	0.222
IM-CD4(H/L)	0.002	0.029 (0.005–0.187)	0.001	0.011	0.077 (0.018–0.329)	0.001
CT-CD8(H/L)	0.114	—	0.320	0.083	—	0.274
IM-CD8(H/L)	0.770	—	0.847	0.795	—	0.452
IS(H/L)	0.005	—	0.121	0.007	0.098 (0.013–0.755)	0.026
CT-PD-L1 (+/−)	0.382	—	0.439	0.245	—	0.375
IM-PD-L1 (+/−)	0.688	—	0.711	0.233	—	0.497

*OS* overall survival, *DFS* disease-free survival, *HR* hazard ratio, *CT* center of tumor, *IM* invasive margin, *IS* immunoscore

### Prognostic value of CD8 and PD-L1 between patients with stage I&II and III&IV dMMR

As IS was previously shown to be a strong indicator of survival [21, 22], it was unexpected that high expression of CD8 at the CT and IM did not improve survival and that the IS failed to show a beneficial effect on OS in multivariate analysis. Considering the loss of survival advantage for patients with stage III&IV dMMR, we hypothesized that the effect of CD8 on prognosis was altered in different stages. Therefore, further subgroup analysis of patients with stage I&II and stage III&IV dMMR was performed.

**Fig. 5:**
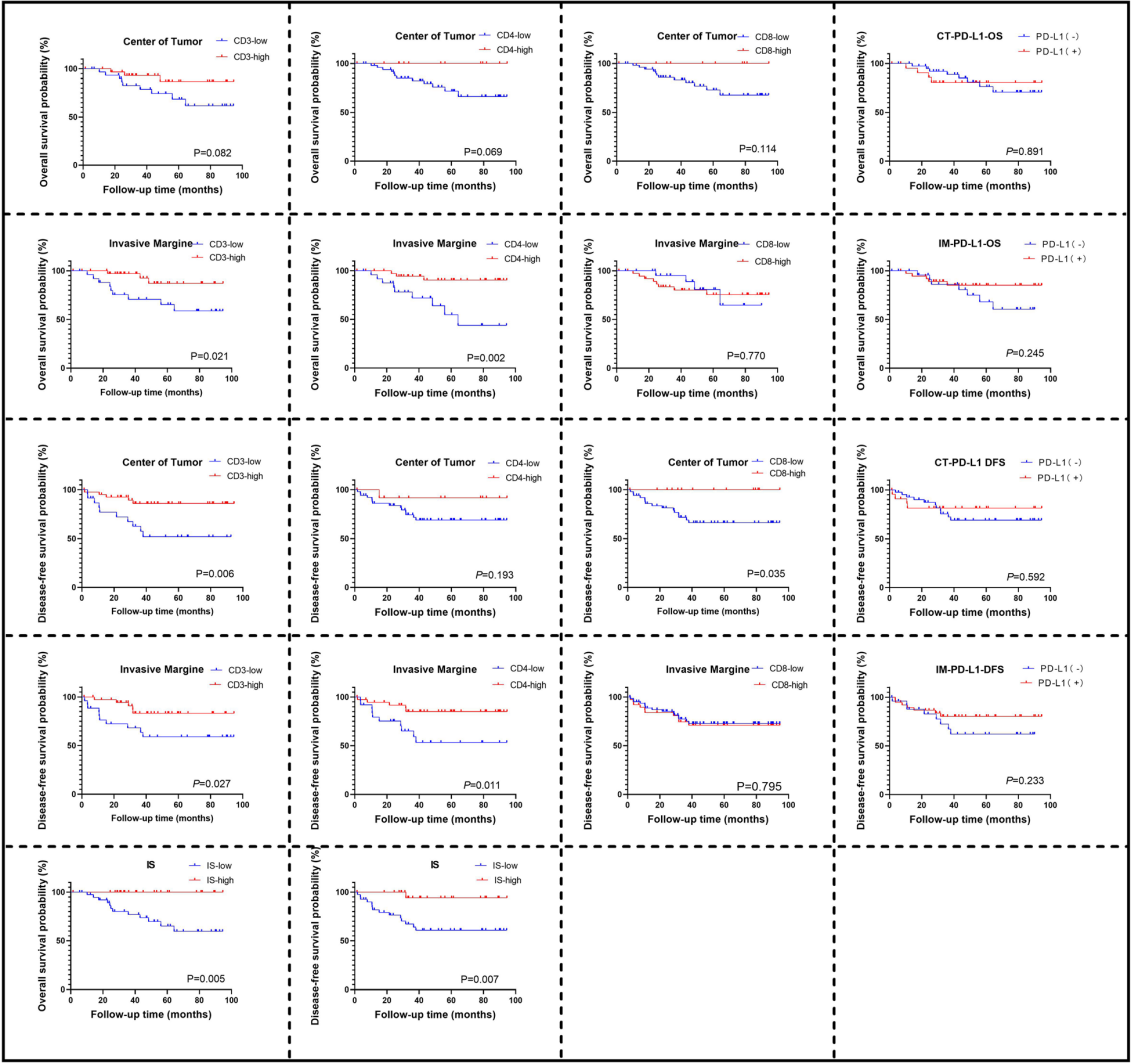
Kaplan-Meier curve of overall survival and disease-free survival between stage I&II and III-IV dMMR patients for expression of CD8 at center of tumor (**a**); CD8 at invasive margin (**b**); PD-L1 at center of tumor (**c**); PD-L1 at invasive margin (**d**)

Although only high expression of CD8 in patients with stage I&II was associated with a significantly better DFS (*P* = 0.039), the difference was not significant in subgroup analysis. Notably, there may be a “reversal” tendency for the prognostic effect on OS and DFS of CD8 and PD-L1 expression at the CT with tumor stage progression (Fig. 5). Subgroup analysis for CD3 and CD4 was also performed, which did not reveal the same tendency (data not shown).

## Discussion

This study explored the prognostic value of dMMR in patients with different stages of CRC as well as the expression and prognostic value of CD3, CD4, CD8, and PD-L1. Our results suggested that stage III&IV dMMR patients showed no survival advantage than stage III&IV pMMR patients. This finding is consistent with that of our previous meta-analysis [10]. In recent years, an increasing number of studies has shown that TILs have a profound influence on cancer survival. The immunoscore, proposed by Galon [18, 22] and based on the expression level of CD3 and CD8 at the CT and IM, showed much higher accuracy for predicting tumor prognosis compared to traditional TNM staging. Our data suggest that there were no significant expression differences between CD3 and CD8 and the calculated IS value between patients with stage I&II and stage III&IV dMMR patients, indicating that TIL levels were similar in the two groups.

As IS was previously shown to be a strong indicator of survival [21, 22], we also explored whether high expression of CD3 and CD8, and a high IS could improve the survival of dMMR patients. However, our results only suggest that expression of CD3 at the CT or IM is an independent risk factor for tumor prognosis, whereas expression of CD8 at the CT or IM region did not show a significant prognostic effect. Moreover, the prognostic value of IS was insignificant in multivariate analysis. These results indicate that the prognostic effect of CD8 differs between tumor stages, thus affecting its prognostic value.

Subgroup analysis of the prognostic value of CD8 expression between stage I&II and III&IV dMMR patients was performed. As expected, the prognostic value of CD8 showed a reversal prognostic effect between patients with stage I&II and stage III&IV dMMR, particularly for expression at the CT. CD8 is expressed in cytotoxic CD8+T cells, which can specifically recognize antigens on antigen-presenting cells; after activation, these cells proliferate, differentiate, and participate in the immune response to attack tumor cells [23]. Malignant tumors can cause effector T cells to lose their antigen recognition, proliferation, and activation functions and to be inhibited by regulatory T cells, resulting in functional loss. This phenomenon is known as T cell exhaustion [24, 25] and is accompanied by the activation of multiple inhibitory molecular receptors such as PD-1/PD-L1 and CTLA4 [17]. The decreased beneficial effect of high CD8 expression on prognosis may be related to tumor immune editing and T cell exhaustion.

No difference in PD-L1 expression was observed between stage I&II and III&IV dMMR patients at the CT or IM. Our results revealed no predictive value for positive PD-L1 expression at the CT or IM. Considering that T cell exhaustion is related to inhibitory checkpoints, subgroup analysis was also performed in different stages to determine the prognostic value of positive PD-L1 expression. Although the results were not significant, the prognostic value of PD-L1 at the CT also showed a potential tendency for reversal of the prognostic effect between patients with stage I&II and III&IV dMMR.

Numerous studies have focused on the prognostic value of positive PD-L1 expression but showed widely variable results. Multiple studies of colorectal cancer have suggested that PD-L1 expression in tumor tissues has no prognostic value, whereas high expression of PD-L1 in TILs can improve tumor prognosis [19, 20, 26]. Some studies reported that high expression of PD-L1 indicates a better prognosis [27]. Li et al. [28] reported that high expression of PD-L1 in TILs predicts a favorable prognosis. However, some studies found that PD-L1 expression had no predictive value [29, 30]. In contrast, a recent study by Ho et al. [31] showed that high expression of PD-L1 in the CT indicate poor prognosis, whereas its high expression in TILs can improve prognosis. These conflicting results may be related to the different PD-L1 antibodies used and limited patient samples [31]. In conclusion, the prognostic value of PD-L1 requires further analysis; if the “survival paradoxical” phenomenon does exist, additional investigations are needed to determine the underlying mechanisms.

High CD3 expression showed excellent prognostic value for predicting better survival and was an independent risk factor. Subgroup analysis revealed no reversal phenomenon. Subgroup analysis for CD4 showed the same results, suggesting that loss of the survival advantage for patients with stage III&IV dMMR may be related to CD8+T cells.

The tumor immune response is performed by antigen-presenting cells, T cells, and B cells. Dendritic cells present TAAs to helper CD4+T cells via the MHC-2 pathway. Helper T cells secrete interferon α, interleukins, and other substances to improve the sensitivity of tumors to toxic T cells [4]. Therefore, we also investigated the prognostic value of CD4 expression. No differences in CD4 expression were found in the CT and IM regions between the two groups. Increased CD4 expression at the IM significantly improved OS and DFS. Previous studies suggested that CD4+ T cells play a central role in initiating and maintaining anti-cancer immune responses [32–34]. Currently, the ability of CD4 expression to predict tumor prognosis is controversial. Studies of pancreatic cancer, oesophageal squamous cell carcinoma, and ovarian cancer showed that high CD4+T cell infiltration can improve prognosis [35–37]. However, some studies reported that increased CD4+T cell infiltration in renal cancer tissues was related to a worse prognosis [38, 39]. Our data suggest that high expression of CD4+T cells at the IM can significantly improve the OS and DFS of CRC patients.

There were some limitations to this study. First, the morbidity of dMMR in all patients with CRC was relatively low, and the morbidity of patients with stage III&IV dMMR was even lower. Only 32 patients had stage III&IV dMMR CRC among 1460 patients. This small sample size may have affected the results of statistical analysis. Additionally, the results of this retrospective study may have been influenced by loss during follow-up, selectivity bias, and other factors.

## Conclusions

In conclusion, our study showed that patients with dMMR had no survival advantage over patients with pMMR in stage III&IV CRC. High CD3 expression at the CT and IM as well as high CD4 expression at the IM showed obvious improvement in OS and DFS. However, high CD8 expression failed to show predictive value for all patients with dMMR, and subgroup analysis revealed an interesting reversal predictive value between patients with early and advanced dMMR, indicating potential functional loss of CD8+T cells in patients with advanced stage dMMR. PD-L1 expression showed no predictive influence on survival but showed a potential trend of reversal predictive value like CD8. The “reversal” phenomenon should be examined in a larger sample size to confirm our results and determine the underlying mechanism, which may be related to T cell exhaustion and activation of inhibitory checkpoints.

## Data Availability

Data is available from the authors by request.

## Abbreviations

dMMR: Deficient mismatch repair
pMMR: Proficient mismatch repair
CRC: Colorectal cancer
MSI-H: Microsatellite instability high
CT: Center of tumor
IM: Invasive margin
OS: Overall survival
DFS: Disease-free survival
TILs: Tumor-infiltrating lymphocytes
TAAs: Tumor-associated antigens
